# Overall survival benefits of cancer drugs in the WHO Model List of Essential Medicines, 2015–2021

**DOI:** 10.1136/bmjgh-2023-012899

**Published:** 2023-09-28

**Authors:** Yue Zhou, Huseyin Naci, Dingyi Chen, Lin Bai, Luwen Shi, Xiaodong Guan, Anita Katharina Wagner

**Affiliations:** 1 Department of Pharmacy Administration and Clinical Pharmacy, School of Pharmaceutical Sciences, Peking University, Beijing, China; 2 Department of Pharmacy, Peking University People's Hospital, Beijing, China; 3 Department of Health Policy, London School of Economics and Political Science, London, UK; 4 International Research Center for Medicinal Administration, Peking University, Beijing, China; 5 Department of Population Medicine, Harvard Medical School and Harvard Pilgrim Health Care Institute, Boston, Massachusetts, USA

**Keywords:** cancer, health policies and all other topics

## Abstract

**Introduction:**

We examined overall survival (OS) benefits for targeted cancer drugs recommended for List of Essential Medicines (EMLs) since 2015. We assessed consistency of decisions in 2019 and 2021 with more specific criteria: OS benefit >4 months and high scores on European Society for Medical Oncology-Magnitude of Clinical Benefit Scale (ESMO-MCBS).

**Methods:**

We identified applications for cancer drug in WHO EMLs from 2015 to 2021. We extracted evidence of OS benefit documented in WHO Technical Report Series (TRS) and compared it to evidence from pivotal trial(s) documented in Food and Drug Administration-approved labels. We retrieved published ESMO-MCBS scores. We summarised availability and magnitude of OS benefit and ESMO-MCBS scores and assessed consistency of inclusion decisions against WHO criteria.

**Results:**

22/54 targeted cancer drug indications were recommended. Among them, 68.2% and 31.8% had OS benefit evidence documented in WHO-TRS and pivotal trials, respectively. Among those not recommended, 59.4% and 56.3% had OS benefit evidence documented in WHO-TRS and pivotal trials, respectively. Of 11 cancer drug indications recommended in 2019 and 2021, 54.5% and 9.1% had evidence of OS benefit >4 months in WHO-TRS and pivotal trials, respectively; 45.5% met ESMO-MCBS criteria. Ten targeted cancer drugs had more than one application for the same indications. Five of those were eventually recommended, including three without new evidence of OS benefit. Additional factors, such as reduced cost, and increased treatment options, seemed to be important factors in the selection.

**Conclusion:**

While WHO has defined approval criteria for cancer drugs EML, we identified areas where adherence of these criteria and communication of the EML approval decision-making processes can be improved.

**WHAT IS ALREADY KNOWN ON THIS TOPIC**

- Studies have evaluated access to and affordability of cancer drugs in the WHO List of Essential Medicines (EML), but there is limited evidence on clinical benefit and adherence to WHO selection criteria of listed cancer drugs.

**WHAT THIS STUDY ADDS**

- From 2015 to 2021, 22 targeted cancer drug indications were recommended for inclusion in the WHO EML.
- For 68.2% (n=15), WHO reviews and 31.8% (n=7), pivotal trials in Food and Drug Administration-approved labels had document overall survival (OS) benefit at the time of EML inclusion decisions.
- Of 11 targeted cancer drug indications recommended for inclusion since implementation of magnitude of benefit criteria in 2019, 54.5% (n=6) and 9.1% (n=1) had evidence of OS benefit >4 months in WHO-Technical Report Series and in pivotal trials, respectively; 45.5% (n=5) met European Society for Medical Oncology-Magnitude of Clinical Benefit Scale criteria.

**HOW THIS STUDY MIGHT AFFECT RESEARCH, PRACTICE OR POLICY**

- Our findings highlight opportunities for improving the application of clinical benefit criteria and for better documenting rationales for cancer drug listings in the WHO EML.

## Introduction

Cancers cause worldwide morbidity and mortality, affecting over 19 million individuals and leading to nearly 10 million deaths in 2020, with a disproportionate death toll in low-income and middle-income countries (LMICs).^1–3^ Over the past half-century, better understanding of the biology of cancers has led to development of new cancer treatments, some of which have greatly improved the survival of cancer patients in high-income countries.^4–7^ The situation differs for patients in LMICs who have limited access to advanced cancer care, including diagnostics, cancer drugs and well-trained personnel, and well-equipped facilities.^8^ In middle-income countries where the services and facilities may exist, access to medicines and opportunities for better outcomes remain limited to those who can pay for the highly-priced treatments.^8^

Since 1977, the WHO publishes and updates every 2 years the List of Essential Medicines (EML). The WHO EML is intended as a guide for countries and regional authorities, especially in low-income and middle-income settings, to design national essential medicines lists for medicines approval, procurement and reimbursement decisions.^9^ The original WHO EML recommended six cancer drugs, and new cancer drugs were added in 1984, 1995 and 1999.^10–12^ Given the discrepancy in cancer burden between high-income and LMICs and advances in the treatment of some cancers in high-income countries, there was a strong call for narrowing the gap in access to cancer drugs worldwide.^13^ Compared with other classes of drugs, the selection process of cancer drugs has been more challenging due to the large volume of newly developed drugs approved rapidly with uncertain benefits and marketed with high and increasing prices. To ensure the clinical benefits of the recommended cancer drugs in EMLs, the WHO has launched a series of evidence-based updates.^14^ In 2014, WHO commissioned the Union for International Cancer Control to undertake a comprehensive review of cancer drugs in the 18th EML published in 2013 and of new medicines proposed for inclusion by researchers and organisations.^15 16^ ‘Meaningful improvements in overall survival (OS) compared with the existing standard of care’ was a criterion for the 2015 additions of new, highly priced targeted cancer drugs.^14^ Different from traditional chemotherapy, target-specific proteins that control cancer cells’ growth and spread.^17^ Targeted cancer drugs constitute the majority of newly approved cancer therapies^18^ and since 2015, an increasing number of cancer drugs have been recommended for inclusion on the WHO EML.^15 19–21^ Magnitude of benefit was one of the criteria considered since the 2015 cancer drug listings^22^ and quantified in 2018 in two metrics: (1) a threshold for OS benefit of at least 4–6 months and (2) a score on the European Society for Medical Oncology-Magnitude of Clinical Benefit Scale (ESMO-MCBS) of A or B in the curative setting and of 4 or 5 in the non-curative setting. These criteria have been recommended for the 2019 and 2021 (21st and 22nd) WHO EMLs.^19^ There is debate about the clinical benefit of new cancer drugs which often are approved based on surrogate outcome measures or on pivotal studies that do not permit inference about clinical benefit.^23–25^ Despite WHO proposed two specific criteria for selecting cancer drugs, lack of fidelity may occur because these are guiding principles for selection, among other criteria. However, WHO’s goal is to list only drugs with meaningful clinical benefit and these adopted guiding principles are important to achieve this goal. To our knowledge, no studies have examined the documented clinical benefit of targeted cancer drugs in the WHO EML or how approval decisions for the latest WHO EMLs align with WHO’s recent magnitude of benefit criteria for selecting cancer drugs. We address these knowledge gaps by assessing documented clinical benefits of WHO-EML cancer drugs. Our specific aims are to (a) assess documented OS benefit for targeted cancer drugs proposed for EML inclusion since 2015 and assess OS benefit magnitude and ESMO-MCBS scores for targeted cancer drugs proposed for listing in the WHO EML since 2019 and (b) assess the consistency of latest listing decisions with WHO criteria for WHO EML cancer drugs.

## Methods

### Data sources

The WHO Technical Report Series (TRS)^15 19–21^ and the WHO electronic EML database^26^ were used to identify the applications for listing of targeted cancer drug indications. The WHO TRS documents were used to retrieve basic information and clinical benefit data documented in EML applications. The Drugs@FDA database^27^ was used to retrieve evidence of OS benefits in pivotal trials and the ESMO-MCBS website^28^ was used to extract ESMO-MCBS scores for indications proposed for listing.

### Study sample

The unit of analysis for this study was the targeted cancer drug indication. We identified applications for targeted cancer drug indications intended for inclusion in the WHO EML based on the final reports of meetings of the WHO expert committee in 2015, 2017, 2019 and 2021, as documented in the WHO Technical Report Series (TRS), Section 8.2.^15 19–21^ Our study period corresponds to the recent increase in the number of targeted cancer medicines considered for listing in the WHO EML. In TRS Section 8.2, applications included not only targeted cancer drug indications, but also cytotoxic medicines, hormones and antihormones, and supportive cancer care medicines. We used the WHO electronic EML database (https://list.essentialmeds.org/) which allowed us to identify eligible applications of targeted cancer drug indications (8.2.2 Targeted therapies and 8.2.3 Immunomodulators). Applications for new formulations of already listed drugs or applications for reinstatement were not included in the analysis.

For each application for listing of targeted cancer drug indications, we extracted relevant information from two parts of the WHO-TRS: (1) ‘Review of benefits and harms’ (2015) or ‘Summary of evidence: benefits (from applicants)’ (2017, 2019 and 2021) and (2) ‘Recommendations’ (2015) or ‘Committee recommendations’ (2017, 2019 and 2021).^15 19–21^ Since clinical benefit data shown in pivotal trials is crucial evidence for supporting the use of cancer drugs, and if it exists, US Food and Drug Administration (FDA) labels list the evidence in pivotal trials, we also gathered this information from the publicly available Drugs@FDA database (https://www.accessdata.fda.gov/scripts/cder/daf/index.cfm). We retrieved the most recent FDA-approved labels at the time of WHO listing decisions and reviewed section 14 ‘CLINICAL STUDIES’ to extract clinical benefit data. We extracted ESMO-MCBS scores based on the trials cited in WHO-TRS from the publicly available ESMO-MCBS website.

### Measures

OS benefit and ESMO-MCBS scores were used as indicators of clinical benefit.

We extracted information on study design (study type, trial group, control group) and OS results by reviewing all references cited in the ‘Review of benefits and harms’ (2015)^15^ or ‘Summary of evidence: benefits (from applicants)’ (2017, 2019 and 2021) of WHO-TRS documents and in section 14 ‘CLINICAL STUDIES’ of FDA approved drug labels. Cancer drug indications with statistically significant OS results were categorised as having documented evidence of OS benefit. We categorised cancer drug indications with unknown or unavailable documented evidence of OS benefit if (1) trial results were not statistically significant, if (2) OS results were not reported or could not be calculated or if (3) the FDA-approved drug label was unavailable, or the drug was not approved by FDA. Based on the trials cited in WHO-TRS, we further extracted the highest score for the proposed indications from ESMO-MCBS website. Cancer drug indications with an ESMO-MCBS score of A or B in the curative setting and of 4 or 5 in the non-curative setting were categorised as meeting the EML selection criterion. We categorised cancer drug indications as not meeting the criterion if (1) the cancer drug indications could not be found on the website, or (2) the trials cited by WHO-TRS were not used by EMSO-MCBS for score evaluation.

### Data analysis

We assessed WHO listing decisions since 2015 with respect to evidence of OS benefit for the cancer drug indications as described in WHO-TRS. We also assessed 2019 and 2021 decisions with respect to evidence of magnitude of OS benefit >4 months (a median gain in OS benefit in the treatment arm of more than 4 months compared with that in the control arm) and ESMO-MCBS scores A or B (curative) or 4 or 5 (non-curative). Then we compared the availability of evidence of OS benefit extracted from WHO-TRS and pivotal trials (as obtained from FDA-approved labels). We noted if one source had documented evidence of OS benefit while the other did not. We then assessed the evidence of OS benefit for the same cancer drug indications which were applied more than once to examine whether new evidence was added in later applications. We further conducted a content analysis to assess how WHO-TRS communicated the evidence supporting listings, especially for those indications that did not have documented evidence of OS benefit. We also noted whether the rationales underlying WHO inclusion decisions were explicitly stated in the ‘Recommendations’ (2015) or ‘Committee recommendations’ (2017, 2019 and 2021) sections, and whether WHO provided a structured summary based on the selection criteria.

We conducted descriptive analyses of cancer drug indication applications across the four most recent WHO EMLs. We further analysed the selection of targeted cancer drug indications in terms of OS benefit based on WHO-TRS and pivotal trials (as reported in FDA-approved drug labels).

### Patient and public involvement

Patients or the public were not involved in the design, conduct, reporting or dissemination plans of our research.

## Results

### WHO EML cancer drug applications and decisions, 2015–2021

From 2015 to 2021, the WHO Expert Committee considered applications for 54 targeted cancer drug indications, of which 40.7% (n=22) were recommended for inclusion in the WHO EML (table 1).

**Table 1:** Applications and recommendations of cancer drug indications, 2015–2021

Year	Targeted cancer drug indication applications (n=54)	
	Recommended, n (%)	Not recommended, n (%)
2015	9 (69.2)	4 (30.8)
2017	2 (28.6)	5 (71.4)
2019	8 (57.1)	6 (42.9)
2021	3 (15.0)	17 (85.0)
Total	22 (40.7)	32 (59.3)

### Clinical benefit of targeted therapy applications

**Figure 1:**
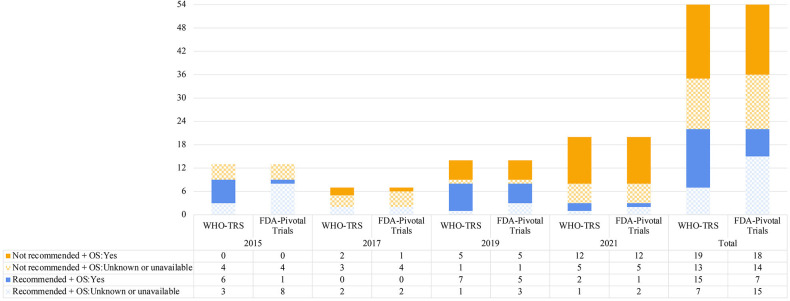
Evidence of overall survival (OS) benefit in applications for targeted cancer drug indications, 2015–2021. FDA-pivotal trials were obtained from FDA-approved labels. FDA-pivotal trials were obtained from FDA-approved labels. FDA-approved labels of all-transretinoic acid (EML decision year 2015) and filgrastim (EML decision year 2015) could not be found. Tislelizumab (EML decision year 2021) is not approved by FDA, and the label is not available. Those corresponding cancer drug indications were categorised as not having documented evidence of OS benefit based on FDA-pivotal trials. EML, List of Essential Medicines; FDA, Food and Drug Administration; WHO-TRS, WHO Technical Report Series.

Figure 1 shows that among the 22 targeted cancer drug indications recommended for inclusion in the 2015–2021 EMLs, 68.2% (n=15) and 31.8% (n=7) had documented evidence of OS benefit in WHO-TRS or in pivotal trials, respectively.

**Figure 2:**
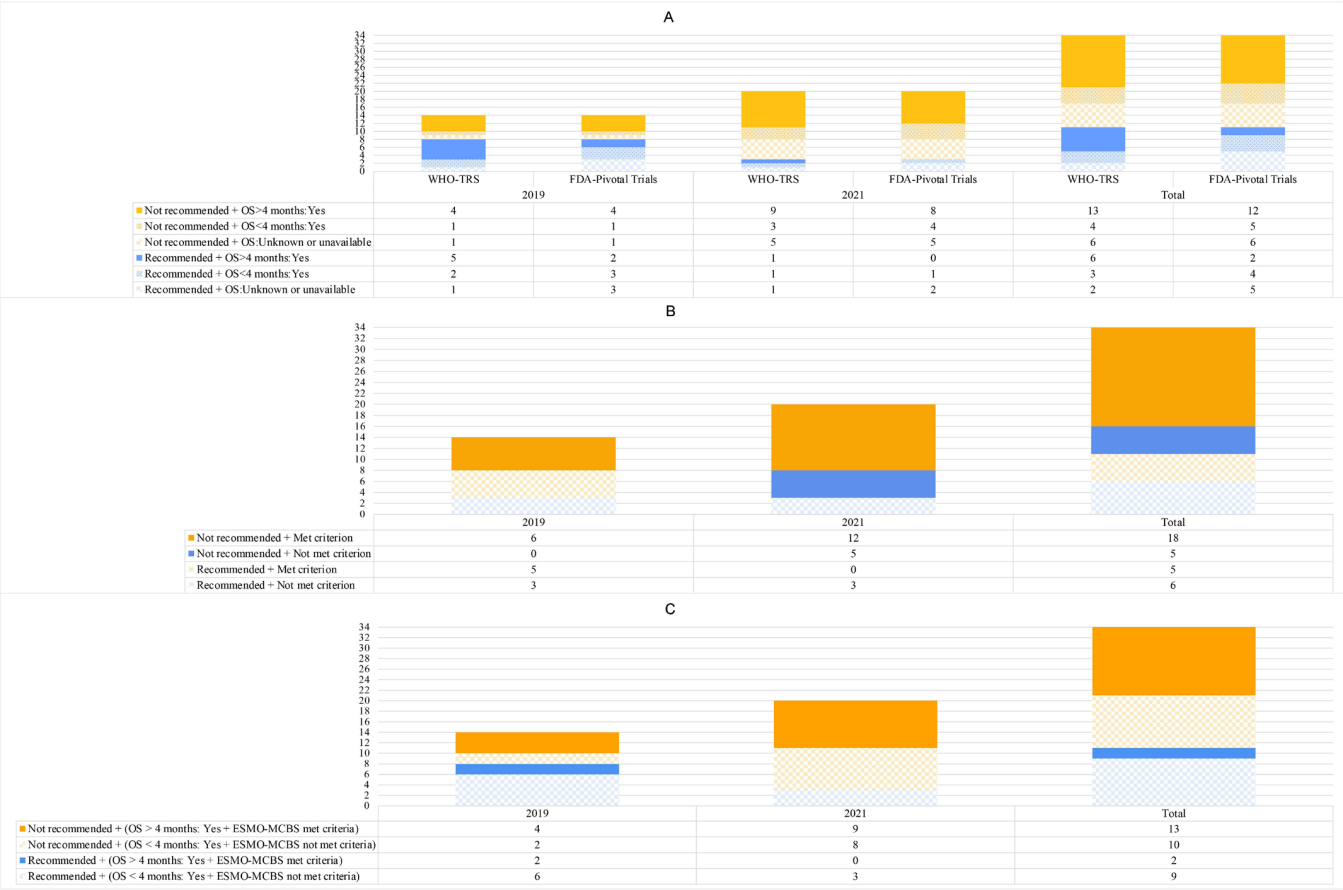
Evidence of overall survival (OS) benefit and scores on ESMO-MCBS in applications for targeted cancer drug indications, 2019–2021. (A) Evidence of OS benefit, 2019–2021; (B) Whether ESMO-MCBS of A or B in the curative setting and of 4 or 5 in the non-curative setting, 2019–2021; (C) Whether Met Both the OS Benefit >4 months and the ESMO-MCBS criteria. FDA-pivotal trials were obtained from FDA approved-labels. Tislelizumab (EML decision year 2021) is not approved by FDA, and the label is not available. Those corresponding cancer drug indications were categorised as not having documented evidence of OS benefit based on FDA-pivotal trials. ESMO-MCBS, European Society for Medical Oncology-Magnitude of Clinical Benefit Scale; FDA, Food and Drug Administration; WHO-TRS, WHO Technical Report Series.

In addition to the criterion of OS benefit evidence in place for the 2015 EML, starting with the 2019 list, WHO defined a clinically meaningful OS benefit as at least a median of 4–6 months and ESMO-MCBS scores of A or B in the curative setting or 4 or 5 in the non-curative setting as EML selection criteria.^19^ Of 11 targeted cancer drug indications recommended for inclusion in the 2019 and 2021 EMLs, 54.5% (n=6) and 9.1% (n=1) had evidence of OS benefit >4 months in WHO-TRS and in pivotal trials, respectively (figure 2A); 45.5% (n=5) met ESMO-MCBS criteria (figure 2B); 18.2% (n=2) met both the OS benefit >4 months and the ESMO-MCBS criteria (figure 2C). Among those meeting the ESMO-MCBS criterion, only nivolumab for metastatic melanoma had a score of ‘A’ in the curative setting. Other indications met the criterion for the non-curative setting (online supplemental etable 1).

10.1136/bmjgh-2023-012899.supp1 Supplementary data

For targeted cancer drug indications that were not recommended (n=23) in the 2019 and 2021 EMLs, we observed that 56.5% (n=13) and 52.2% (n=12) had documented evidence of OS benefit >4 months in WHO-TRS and in pivotal trials, respectively (figure 2A); 78.3% (n=18) met the ESMO-MCBS score criterion (figure 2B); 56.5% (n=13) met both the OS benefit >4 months and the ESMO-MCBS criteria (figure 2C). Evidence of OS benefit in application for targeted cancer drug indications from 2015 to 2021 is shown in online supplemental efigure 1.

**Figure 3:**
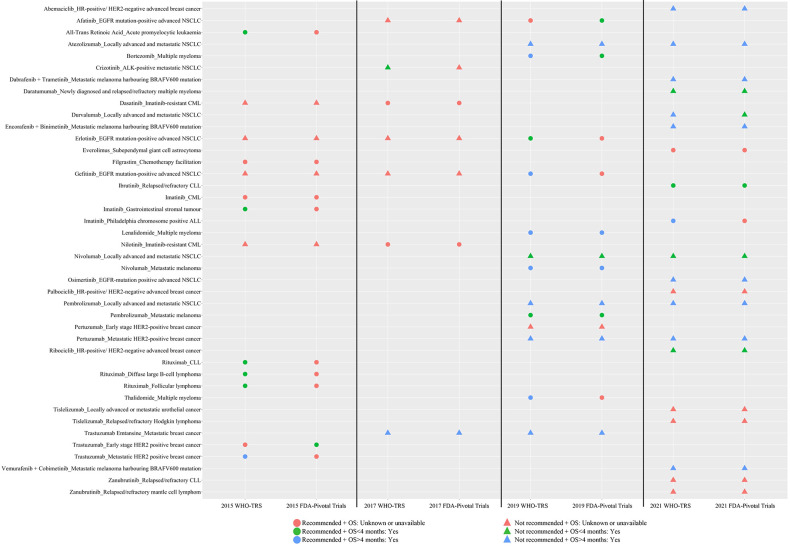
Comparison of documented evidence of OS benefit for 54 targeted cancer drug indications in WHO technical report series and pivotal trials reported in US FDA-Approved Labels, 2015–2021. OS result was not reported in WHO-TRS documents for Trastuzumab-Early stage HER2 positive breast cancer. FDA-approved labels of all-transretinoic acid (EML decision year 2015) and filgrastim (EML decision year 2015) could not be found. Tislelizumab (EML decision year 2021) is not approved by FDA, and the label is not available. For the targeted cancer drugs with FDA-approved labels available, OS results were not reported for rituximab-CLL, rituximab-follicular lymphoma, trastuzumab-Metastatic HER2 positive breast cancer, gefitinib-EGFR mutation-positive advanced NSCLC. (1) Primary prophylaxis in patients at high risk for developing febrile neutropenia associated with myelotoxic chemotherapy, (2) secondary prophylaxis for patients who have experienced neutropenia following prior myelotoxic chemotherapy, (3) to facilitate administration of dose dense chemotherapy regimens. ALK, anaplastic lymphoma kinase; ALL, acute lymphoblastic leukaemia; CLL, chronic lymphocytic leukaemia; CML, chronic myeloid leukaemia; EGFR, epidermal growth factor receptor; EML, List of Essential Medicines; FDA, Food and Drug Administration; HER2, human epidermal growth factor receptor 2; HR, hormone receptor; NSCLC, non-small-cell lung carcinoma; OS, overall survival; WHO-TRS, WHO Technical Report Series.

Ten targeted cancer drugs had more than one application for the same indications over several application cycles and five were eventually recommended for inclusion in the WHO EML (figure 3 and table 2). Among the recommended targeted cancer drug indications, only gefitinib for EGFR mutation-positive advanced non-small cell lung cancer (NSCLC) met the WHO EML OS benefit criterion. Compared with documentation in the 2015 WHO-TRS, new OS benefit evidence was provided for erlotinib for treatment of EGFR mutation-positive advanced NSCLC in 2019 WHO-TRS; however, OS benefit was less than 4 months. Repeated applications for the other recommended targeted cancer drug indications did not provide new evidence of OS benefit.

**Table 2:** OS benefit of targeted cancer drugs with more than one application

Year	Medicine	Indication	Eventually recommended	New OS benefit evidence in repeat applications
2015	Dasatinib	Imatinib-resistant CML	Yes	No
2017				
2015	Nilotinib	Imatinib-resistant CML	Yes	No
2017				
2017	Afatinib	EGFR mutation-positive advanced NSCLC	Yes	No
2019				
2015	Erlotinib	EGFR mutation-positive advanced NSCLC	Yes	Yes (OS benefit <4 months)
2017				
2019				
2015	Gefitinib	EGFR mutation-positive advanced NSCLC	Yes	Yes (OS>4 months)
2017				
2019				
2017	Trastuzumab emtansine	Metastatic breast cancer	No	No
2019				
2019	Pertuzumab	Metastatic HER2-positive breast cancer	No	Yes (OS >4 months)
2021				
2019	Atezolizumab	Locally advanced and metastatic NSCLC	No	No
2021				
2019	Nivolumab	Locally advanced and metastatic NSCLC	No	No
2021				
2019	Pembrolizumab	Locally advanced and metastatic NSCLC	No	No
2021				

CML, chronic myeloid leukaemia; NSCLC, non-small cell lung cancer; OS, overall survival.

For 13 targeted cancer drug indications, availability of evidence of OS benefit differed between WHO-TRS and pivotal trials (as reported in FDA-approved drug labels) (figure 2, online supplemental etable 1 and efigure 2). For 11 indications, evidence of OS benefit was only documented in WHO-TRS; for two indications, documented evidence of OS benefit was only found in pivotal trials. Conflicting OS benefit evidence was observed for four targeted cancer drug indications. Discrepancies were due to different evidence sources (different trials, meta-analysis vs trial, retrospective study vs trial, review vs trial) and treatment comparators.

Seven targeted cancer drug indications without evidence of OS benefit were recommended for EML inclusion (table 3). Additional factors, such as reduced cost and increased treatment options, seemed to be more important than OS benefits or ESMO-MCBS scores in the selection for the WHO EMLs.

**Table 3:** Decision rationales for recommendations of cancer drug indications without OS benefit evidence

EML year	Drug and indication	OS benefit documented in WHO-TRS	Decision rationales (as worded in WHO TRS documents)	Type of WHO-TRS decision rationale
2015	Filgrastim-Chemotherapy facilitation	No	‘Several studies have shown the comparability in effectiveness and patient outcomes of daily filgrastim and once per cycle pegfilgrastim (532–534). A meta analysis in 2007, analysing outcomes among patients with different types of cancer (and different chemotherapy regimens), concluded that pegfilgrastim produced moderately better outcomes than filgrastim (535).’	Comparative effectiveness; increasing treatment options; biosimilar availability; lower cost
			‘In general, however, the choice between filgrastim and pegfilgrastim largely concerns individual clinical preference, ease of administration and the difference in cost; pegfilgrastim is much more expensive than filgrastim. Additionally, biosimilars are available for filgrastim, allowing for comparable clinical efficacy at lower cost. Guidelines are generally accepting of both options, depending on patient circumstances and cost considerations within the health system concerned (536).’	
2015	Imatinib-CML	No	‘On the basis of the evidence presented…’ (referenced WSR evidence unclear)	N/A
2015	Trastuzumab-Early stage HER2 positive breast cancer	No	‘On the basis of the evidence presented in the application’ (referenced WSR evidence unclear)	N/A
2017	Dasatinib-Imatinib-resistant CML	No	‘Despite these shortcomings, the Expert Committee considered that nilotinib and dasatinib have been shown to be valid treatment options for use in patients with chronic myeloid leukaemia and imatinib resistance.’	Relevant benefit; increasing treatment options
			‘Considering all relevant clinical outcomes, the Committee accepted that there is a relevant clinical benefit resulting primarily from large response rates (ie, complete cytogenetic response) in patients with otherwise very limited treatment options (eg, donor stem cell transplant).’	
2017	Nilotinib-Imatinib-resistant CML	No	‘Despite these shortcomings, the Expert Committee considered that nilotinib and dasatinib have been shown to be valid treatment options for use in patients with chronic myeloid leukaemia and imatinib resistance.’	Relevant benefit; increasing treatment options
			‘Considering all relevant clinical outcomes, the Committee accepted that there is a relevant clinical benefit resulting primarily from large response rates (ie, complete cytogenetic response) in patients with otherwise very limited treatment options (eg, donor stem cell transplant).’	
2019	Afatinib-EGFR mutation-positive advanced NSCLC	No	‘The Committee noted that these medicines are associated with relevant survival benefits for patients, acceptable toxicity and improvements in quality of life compared with chemotherapy.’	Relevant benefit; generic availability; diagnostic test availability
			‘The Committee also noted that since these medicines were considered for inclusion on the EML in 2015, generic versions of these medicines are more widely available, as are quality-assured diagnostic molecular tests for EGFR mutations.’	
2021	Everolimus-Subependymal giant cell astrocytoma	No	‘The Expert Committee noted that subependymal giant cell astrocytoma (SEGA) is a rare disease affecting almost exclusively children with tuberous sclerosis complex and is associated with considerable neurological morbidity and mortality.’	Increasing treatment options
			‘SEGA management historically had few options other than surgery, as radiotherapy and chemotherapy were not effective.’	

Chemotherapy facilitation, (1) primary prophylaxis in patients at high risk for developing febrile neutropenia associated with myelotoxic chemotherapy, (2) secondary prophylaxis for patients who have experienced neutropenia following prior myelotoxic chemotherapy, (3) to facilitate administration of dose dense chemotherapy regimens.

CML, chronic myeloid leukaemia; EGFR, epidermal growth factor receptor; EML, Model List of Essential Medicines; HER2, human epidermal growth factor receptor 2; NSCLC, non-small-cell lung carcinoma; OS, overall survival; WHO-TRS, WHO Technical Report Series.

## Discussion

We find that across the four most recent WHO EMLs, about one-third of the recommended targeted cancer drug indications lacked the evidence of OS benefit, as indicated by WHO-TRS not reporting or reporting non-significant OS data. The proportion increased to two-thirds when based on OS benefit evidence available in pivotal trials underlying FDA drug approvals alone. Our results point to inconsistencies in the WHO selection of essential cancer drugs against a desired clinical benefit criterion defined as OS benefit. We also report discrepancies between OS benefit results documented in WHO-TRS and pivotal trials documented in FDA-approved labels.

Selection of cancer drug indications for the WHO EML is complex. In addition to clinical efficacy, the EML Committee is tasked with considering non-clinical factors including burden of disease, safety, availability of alternative treatment options and cost (both to the health system and individual patients). Of concern are potential barriers to access to and affordability of essential cancer drugs recommended in the WHO EML.^29–35^ Arguably, access and affordability are only relevant considerations for WHO EML cancer drugs with established clinical benefit, and most importantly, OS benefit. In recent years, the WHO has put greater emphasis on the development and use of explicit clinical benefit criteria to inform the selection of cancer drugs for the EML.

Indeed, WHO has regarded OS benefit as one of the fundamental criteria for essential cancer medicine selection since 2015.^15^ In 2018, WHO identified a threshold for OS benefit of at least 4–6 months for all cancer drug indications under consideration.^19^ During our study period, we observed that the OS benefit criterion was implemented inconsistently. Across the 2015–2021 EMLs, 15/22 listed targeted cancer drug indications had evidence of OS. Of 11 targeted cancer drug indications recommended for inclusion in the 2019 and 2021 EMLs, WHO-TRS reported evidence of median OS benefit >4 months for six. Of five cancer drug indications that sought inclusion in the WHO EML more than once, three were subsequently recommended without new data on OS benefit. A relatively high proportion of drug indications with OS benefit >4 months were not recommended. Similarly, all the not-recommended cancer drug indications met the ESMO-MCBS criterion. Our findings suggest that OS benefit and ESMO-MCBS scores may not always be the primary factor in the decision-making process for EML drug selection. For some cancer drug indications without documented evidence of OS benefit or not meeting ESMO-MCBS score criteria, WHO appears to have placed more emphasis on factors other than clinical benefit for inclusion in the EML.

As the USA leads the world in new drug research and development and is primarily the first market to launch new cancer drugs, many LMICs rely on a drug’s FDA approval status to inform its use in their populations.^36^ In addition, the clinical trials considered by the FDA are often the only studies available evaluating the efficacy of new cancer drugs. We compared the documented evidence of OS benefit between WHO-TRS and pivotal trials reported in FDA labels and found that benefit evidence differed. The proportion of cancer drug indications recommended without documented OS benefit evidence was higher when based on pivotal trial evidence in FDA-approved labels compared with evidence documented in WHO-TRS. These differences were primarily attributable to different sources of OS benefit evidence documented in WHO-TRS and FDA-approved labels. WHO-TRS includes OS benefit information from a wider range of sources, including trials, reviews and retrospective studies, while pivotal clinical trials form the basis of OS benefit evidence in FDA-approved labels. Although the WHO may include follow-up studies that were not included in FDA labels, our findings based on ESMO-MCBS also showed that more than half of the cancer drug indications recommended in 2019 and 2021 lacked ‘clinical meaningful benefit’. In 2018, WHO proposed that availability of evidence from clinical trials, especially high-quality randomised controlled trials, was an important consideration in cancer drug selection decisions.^19^ However, our finding highlights opportunities for greater adherence to this important recommended standard for recommending cancer drugs and the need to further formulate standards for evidence sources of OS benefits used for EML cancer drug selection.

There are important opportunities for more effectively communicating the evidence to support EML selections, as well as the Committee’s rationales for decisions. First, we suggest a more structured and comprehensive reporting of evidence that WHO assembles for EML listing decisions. Research has shown that structured formats for presenting clinical trial information can improve understanding and comprehension of end users.^37^ In terms of efficacy, a tabular reporting format may include (a) the source of OS benefit information (ie, whether it was obtained from a randomised controlled trial, meta-analysis of multiple randomised controlled trials, or retrospective analyses), (b) the quality of OS benefit information (ie, risk of trial bias), (c) availability of evidence of OS benefit (yes/no), (d) magnitude of OS benefit ≥4 months (yes/no) and (e) characteristics of populations in which OS benefit was documented. WHO may also more clearly label the cancer drugs without evidence of documented OS benefit at the time of listing to inform decision-makers. Second, the WHO selection committee may make its decision rationales more accessible by consistently reporting whether decisions were driven by (a) clinical efficacy evidence, (b) comparative safety profiles, (c) expected ease of drug administration and/or (d) cost considerations for LMICs,^38 39^ or other factors.

Our study has several limitations. First, we evaluated documentation of OS benefit evidence in WHO-TRS and FDA-approved labels and did not evaluate the quality of the evidence. WHO also adopted, starting with the 2019 EML, criteria for quality of cancer drug trials.^14^ Since quality of cancer drug trials varies, and poor quality trials may overestimate OS benefit of cancer drugs,^40^ we may have overestimated adherence of EML selections to the most recent selection criteria. Second, no additional published evidence, such as follow-up studies, was included. This would have been particularly interesting in cases where the study endpoint median OS was not reached. However, the focus of the study was to examine the clinical benefit of cancer drug indications at the time of EML selection. Third, we retrieved ESMO-MCBS scores based on the trials cited in WHO-TRS documents which were also used for evaluation by ESMO. This may underestimate the clinical benefit of the drug indications. Fourth, we do not address public health relevance and safety which depend on local circumstances. Finally, we only focus on WHO EML cancer drugs for adults. Further studies should also evaluate selection of cancer drugs for the WHO EML for children.

## Conclusion

In conclusion, the WHO EML is designed to support health system decision-makers, particularly in resource-limited settings, in prioritising medicines for regulatory approval, procurement and financing.^9^ Since 2015, more targeted cancer drugs have been recommended for inclusion in the WHO EML. Given limited evidence of clinical benefit of new targeted cancer drugs, WHO laudably defined criteria for clinical benefit evidence for cancer drug inclusion in the EML. Our findings highlight opportunities for improving application of these desirable criteria and for better documenting the evidence considered and rationales for WHO EML selection decisions.

## Data Availability

Data are available in a public, open access repository. Data supporting the findings were collected from publicly available data (WHO-TRS, FDA-approved labels, ESMO-MCBS).

## References

[1] Global Health Metrics. Global burden of disease study 2019-Neoplasms. n.d. Available: https://www.thelancet.com/pb-assets/Lancet/gbd/summaries/diseases/neoplasms.pdf

[2] Swann T. “'anarchist Technologies': anarchism, cybernetics and mutual aid in community responses to the COVID-19 crisis”. Organization (Lond) 2023;30:193–209. 10.1177/13505084221090632 37038431 PMC10076238

[3] Cancer today-cancer fact sheets. 2020. Available: https://gco.iarc.fr/today/data/factsheets/cancers/39-All-cancers-fact-sheet.pdf

[4] DeVita VT, Chu E. A history of cancer chemotherapy. Cancer Res 2008;68:8643–53. 10.1158/0008-5472.CAN-07-6611 18974103

[5] DeSantis CE, Ma J, Gaudet MM, et al. Breast cancer statistics, 2019. CA Cancer J Clin 2019;69:438–51. 10.3322/caac.21583 31577379

[6] European Commission. European cancer information system. n.d. Available: https://ecis.jrc.ec.europa.eu/factsheets.php

[7] Sledge GW. What is targeted therapy J Clin Oncol 2005;23:1614–5. 10.1200/JCO.2005.01.016 15755966

[8] Farmer P, Frenk J, Knaul FM, et al. Expansion of cancer care and control in countries of low and middle income: a call to action. Lancet 2010;376:1186–93. 10.1016/S0140-6736(10)61152-X 20709386

[9] Selection of essential medicines at country level. Using the WHO model list of essential medicines to update a national essential medicines list, Available: https://www.who.int/publications-detail-redirect/9789241515443

[10] Essential drugs for cancer chemotherapy: memorandum from a WHO meeting. Bull World Health Organ 1985;63:999–1002. 3879680 PMC2536475

[11] WHO Consultation. Essential drugs for cancer chemotherapy. Bull World Health Organ 1994;72:693–8. 7525093 PMC2486570

[12] Sikora K, Advani S, Koroltchouk V, et al. Essential drugs for cancer therapy: a world health organization consultation. Annals of Oncology 1999;10:385–90. 10.1023/A:1008367822016 10370779

[13] Robertson J, Magrini N, Barr R, et al. Medicines for cancers in children: the WHO model for selection of essential medicines. Pediatr Blood Cancer 2015;62:1689–93. 10.1002/pbc.25564 25929524 PMC5132122

[14] Jenei K, Aziz Z, Booth C, et al. Cancer medicines on the WHO model list of essential medicines: processes, challenges, and a way forward. Lancet Glob Health 2022;10:e1860–6. 10.1016/S2214-109X(22)00376-X 36183737

[15] Flatt VD, Campos CR, Kraemer MP, et al. Compositional variation and Bioactivity of the leaf essential oil of Montanoa Guatemalensis from Monteverde, Costa Rica: A preliminary investigation. Medicines (Basel) 2015;2:331–9. 10.3390/medicines2040331 Available: https://www.who.int/publications-detail-redirect/9789241209946 28930215 PMC5456209

[16] Shulman LN, Wagner CM, Barr R, et al. Proposing essential medicines to treat cancer: Methodologies, processes, and outcomes. J Clin Oncol 2016;34:69–75. 10.1200/JCO.2015.61.8736 26578613 PMC5070565

[17] National Cancer Institute. n.d. Targeted therapy for cancer - NCI. Available: https://www.cancer.gov/about-cancer/treatment/types/targeted-therapies

[18] IQVIA. Global oncology trends 2022 - IQVIA. n.d. Available: https://www.iqvia.com/insights/the-iqvia-institute/reports/global-oncology-trends-2022

[19] WHO. The selection and use of essential medicines) - TRS 1021. 2019. Available: https://www.who.int/publications-detail-redirect/9789241210300

[20] WHO. The selection and use of essential medicines - TRS 1006. 2017. Available: https://www.who.int/publications-detail-redirect/9789241210157

[21] WHO. The selection and use of essential medicines - TRS 1035. 2021. Available: https://www.who.int/publications-detail-redirect/9789240041134

[22] Mayor S. WHO includes 16 new cancer drugs on list of essential medicines. Lancet Oncol 2015;16:S1470-2045(15)70240-8. 10.1016/S1470-2045(15)70240-8 26004373

[23] Davis C, Naci H, Gurpinar E, et al. Availability of evidence of benefits on overall survival and quality of life of cancer drugs approved by European medicines agency: retrospective cohort study of drug approvals 2009-13. BMJ 2017;359:j4530. 10.1136/bmj.j4530 28978555 PMC5627352

[24] Chen EY, Haslam A, Prasad V. FDA acceptance of Surrogate end points for cancer drug approval: 1992-2019. JAMA Intern Med 2020;180:912–4. 10.1001/jamainternmed.2020.1097 32338703 PMC7186918

[25] Gyawali B, Hey SP, Kesselheim AS. Assessment of the clinical benefit of cancer drugs receiving accelerated approval. JAMA Intern Med 2019;179:906–13. 10.1001/jamainternmed.2019.0462 31135808 PMC6547118

[26] eEML - Electronic Essential Medicines List, Available: https://list.essentialmeds.org/

[27] Drugs@FDA: FDA-Approved Drugs, Available: https://www.accessdata.fda.gov/scripts/cder/daf/index.cfm

[28] ESMO. ESMO-MCBS for Solid Tumours, Available: https://www.esmo.org/guidelines/esmo-mcbs/esmo-mcbs-for-solid-tumours

[29] Robertson J, Barr R, Shulman LN, et al. Essential medicines for cancer: WHO recommendations and national priorities. Bull World Health Organ 2016;94:735–42. 10.2471/BLT.15.163998 27843163 PMC5043203

[30] Cherny NI, Sullivan R, Torode J, et al. ESMO international consortium study on the availability, out-of-pocket costs and accessibility of antineoplastic medicines in countries outside of Europe. Ann Oncol 2017;28:2633–47. 10.1093/annonc/mdx521 28950323 PMC5834140

[31] Chivukula MV, Tisocki K. Approaches to improving access to essential cancer medicines in the WHO South-East Asia region. WHO South East Asia J Public Health 2018;7:62–6. 10.4103/2224-3151.239415 30136662

[32] Martei YM, Chiyapo S, Grover S, et al. Availability of WHO essential medicines for cancer treatment in Botswana. J Glob Oncol 2018;4:1–8. 10.1200/JGO.17.00063 PMC6223417 30241225

[33] Baxi SM, Beall R, Yang J, et al. A Multidisciplinary review of the policy, intellectual property rights, and international trade environment for access and Affordability to essential cancer medications. Global Health 2019;15:57. 10.1186/s12992-019-0497-3 31533850 PMC6751842

[34] Ferrario A, Stephens P, Guan X, et al. Sales of anti-cancer medicines; China, Indonesia, Kazakhstan, Malaysia, Philippines and Thailand. Bull World Health Organ 2020;98:467–74. 10.2471/BLT.19.243998 32742032 PMC7375213

[35] Chen C, Feng Z, Ding Y, et al. What factors hindered the access to essential anticancer medicine in public hospitals for the local population in Hubei province, China. Front Pharmacol 2021;12:734637. 10.3389/fphar.2021.734637 34630110 PMC8499033

[36] Akhade A, Sirohi B, Gyawali B. Global consequences of the US FDA's accelerated approval of cancer drugs. Lancet Oncol 2022;23:201–3. 10.1016/S1470-2045(21)00709-9 35114117

[37] Schwartz LM, Woloshin S. The drug facts box: improving the communication of prescription drug information. Proc Natl Acad Sci U S A 2013;110:14069–74. 10.1073/pnas.1214646110 23942130 PMC3752172

[38] Moucheraud C, Wirtz VJ, Reich MR. Evaluating the quality and use of economic data in decisions about essential medicines. Bull World Health Organ 2015;93:693–9. 10.2471/BLT.14.149914 26600611 PMC4645430

[39] Eniu A, Cherny NI, Bertram M, et al. Cancer medicines in Asia and Asia-Pacific: what is available, and is it effective enough ESMO Open 2019;4:e000483. 10.1136/esmoopen-2018-000483 31423334 PMC6677966

[40] Naci H, Davis C, Savović J, et al. Design characteristics, risk of bias, and reporting of randomised controlled trials supporting approvals of cancer drugs by European medicines agency, 2014-16: cross sectional analysis. BMJ 2019;366:l5221. 10.1136/bmj.l5221 31533922 PMC6749182

