# Supplementary material for: Overall survival benefits of cancer drugs in the WHO Model List of Essential Medicines, 2015–2021
Source: BMJ Glob Health. 2023 Sep 28;8(9):e012899. doi: 10.1136/bmjgh-2023-012899 (PMC10546158; doi:10.1136/bmjgh-2023-012899)
Supplement: Supplementary data [file bmjgh-2023-012899supp001.pdf]

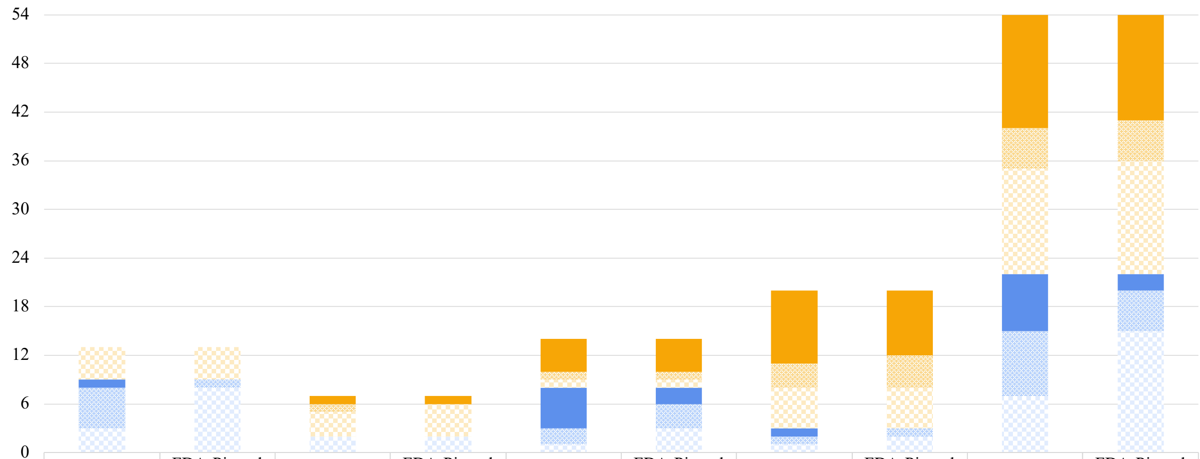

	2015		2017		2019		2021		Total	
	WHO-TRS	FDA-Pivotal Trials	WHO-TRS	FDA-Pivotal Trials	WHO-TRS	FDA-Pivotal Trials	WHO-TRS	FDA-Pivotal Trials	WHO-TRS	FDA-Pivotal Trials
Not recommended + OS>4 months:Yes	0	0	1	1	4	4	9	8	14	13
Not recommended + OS<4 months:Yes	0	0	1	0	1	1	3	4	5	5
Not recommended + OS:Unknown or unavailable	4	4	3	4	1	1	5	5	13	14
Recommended + OS>4 months:Yes	1	0	0	0	5	2	1	0	7	2
Recommended + OS<4 months:Yes	5	1	0	0	2	3	1	1	8	5
Recommended + OS:Unknown or unavailable	3	8	2	2	1	3	1	2	7	15

eFigure 1 Evidence of Overall Survival Benefit in Applications for Targeted Cancer Drug Indications, 2015-2021.

FDA-pivotal trials were obtained from FDA approved-labels. FDA-pivotal trials were obtained from FDA approved-labels. FDA-approved labels of All-trans Retinoic Acid (EML decision year 2015) and Filgrastim (EML decision year 2015) could not be found. Tislelizumab (EML decision year 2021) is not approved by FDA, and the label is not available. Those corresponding cancer drug indications were categorized as not having documented evidence of OS benefit based on FDA-pivotal trials.

WHO-TRS, WHO Technical Report Series; OS, overall survival.

Note: WHO EML selection criteria for 2015 and 2017 EMLs included "meaningful clinical benefit"; for 2019 and 2021 EMLs, selection criteria included overall survival benefit >4-6 months. eFigure1 presents the most recent overall survival benefit criteria for all 54 indication applications for the 2017-2021 EMLs.

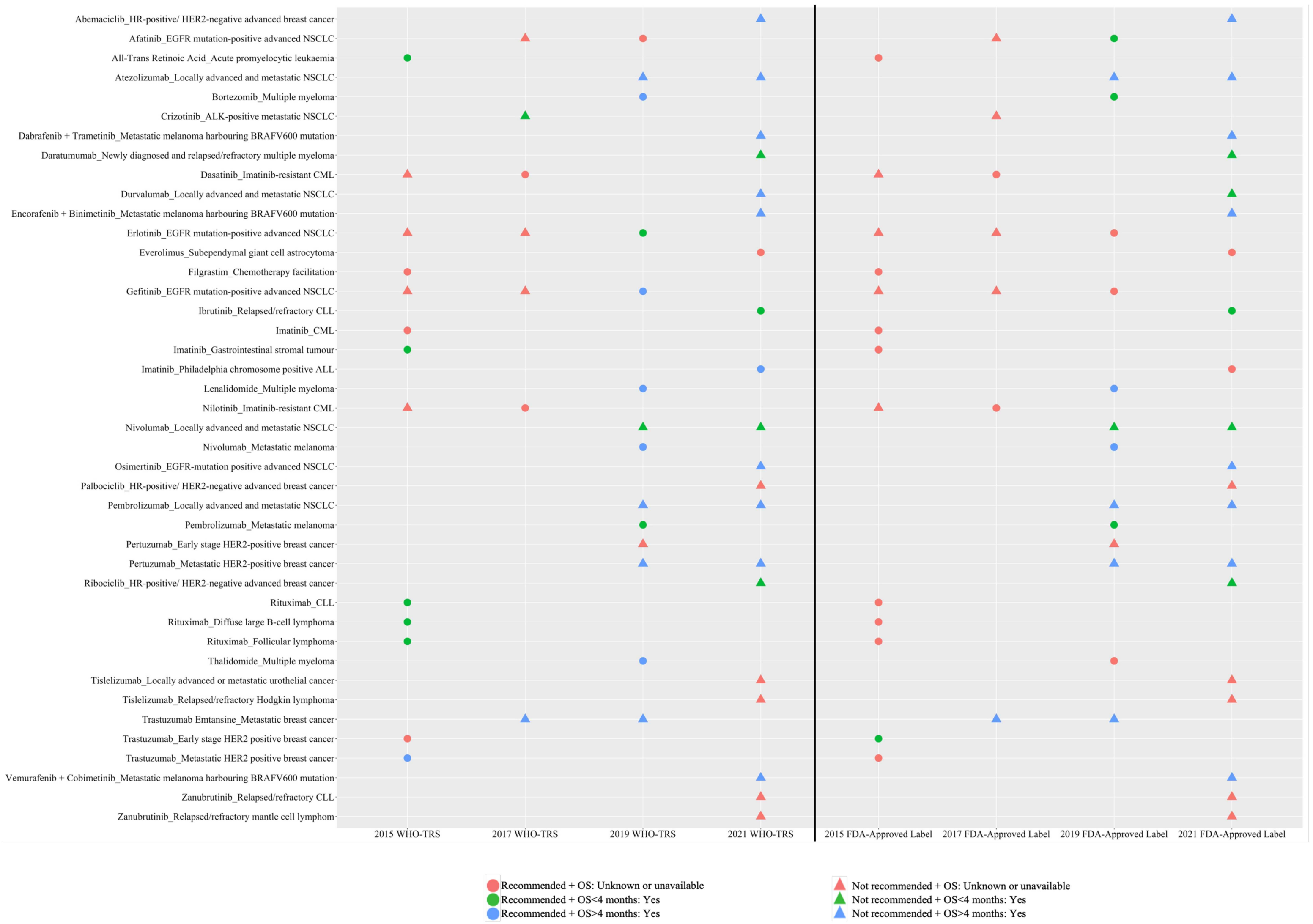
Abemaciclib_HR-positive/ HER2-negative advanced breast cancer
Afatinib_EGFR mutation-positive advanced NSCLC
All-Trans Retinoic Acid_Acute promyelocytic leukaemia
Atezolizumab_Locally advanced and metastatic NSCLC
Bortezomib_Multiple myeloma
Crizotinib_ALK-positive metastatic NSCLC
Dabrafenib + Trametinib_Metastatic melanoma harbouring BRAFV600 mutation
Daratumumab_Newly diagnosed and relapsed/refractory multiple myeloma
Dasatinib_Imatinib-resistant CML
Durvalumab_Locally advanced and metastatic NSCLC
Encorafenib + Binimetinib_Metastatic melanoma harbouring BRAFV600 mutation
Erlotinib_EGFR mutation-positive advanced NSCLC
Everolimus_Subependymal giant cell astrocytoma
Filgrastim_Chemotherapy facilitation
Gefitinib_EGFR mutation-positive advanced NSCLC
Ibrutinib_Relapsed/refractory CLL
Imatinib_CML
Imatinib_Gastrointestinal stromal tumour
Imatinib_Philadelphia chromosome positive ALL
Lenalidomide_Multiple myeloma
Nilotinib_Imatinib-resistant CML
Nivolumab_Locally advanced and metastatic NSCLC
Nivolumab_Metastatic melanoma
Osimertinib_EGFR-mutation positive advanced NSCLC
Palbociclib_HR-positive/ HER2-negative advanced breast cancer
Pembrolizumab_Locally advanced and metastatic NSCLC
Pembrolizumab_Metastatic melanoma
Pertuzumab_Early stage HER2-positive breast cancer
Pertuzumab_Metastatic HER2-positive breast cancer
Ribociclib_HR-positive/ HER2-negative advanced breast cancer
Rituximab_CLL
Rituximab_Diffuse large B-cell lymphoma
Rituximab_Follicular lymphoma
Thalidomide_Multiple myeloma
Tislelizumab_Locally advanced or metastatic urothelial cancer
Tislelizumab_Relapsed/refractory Hodgkin lymphoma
Trastuzumab Emtansine_Metastatic breast cancer
Trastuzumab_Early stage HER2 positive breast cancer
Trastuzumab_Metastatic HER2 positive breast cancer
Vemurafenib + Cobimetinib_Metastatic melanoma harbouring BRAFV600 mutation
Zanubrutinib_Relapsed/refractory CLL
Zanubrutinib_Relapsed/refractory mantle cell lymphom
2015 WHO-TRS
2017 WHO-TRS
2019 WHO-TRS
2021 WHO-TRS
2015 FDA-Approved Label
2017 FDA-Approved Label
2019 FDA-Approved Label
2021 FDA-Approved Label
Recommended + OS: Unknown or unavailable
Recommended + OS<4 months: Yes
Recommended + OS>4 months: Yes
Not recommended + OS: Unknown or unavailable
Not recommended + OS<4 months: Yes
Not recommended + OS>4 months: Yes

BMJ Publishing Group Limited (BMJ) disclaims all liability and responsibility arising from any reliance placed on this supplemental material which has been supplied by the author(s)

eFigure 2 EML Recommendation Decisions and Documented Evidence of OS Benefit for 54 Targeted Cancer Drug Indications, 2015-2021.
FDA-pivotal trials were obtained from FDA approved-labels. FDA-pivotal trials were obtained from FDA approved-labels. FDA-approved labels of All-trans Retinoic Acid (EML decision year 2015) and Filgrastim (EML decision year 2015) could not be found. Tislelizumab (EML decision year 2021) is not approved by FDA, and the label is not available. Those corresponding cancer drug indications were categorized as not having documented evidence of OS benefit based on FDA-pivotal trials.
OS, Overall survival; ALK, anaplastic lymphoma kinase; ALL, Acute lymphoblastic leukaemia; CLL, Chronic lymphocytic leukaemia; CML, Chronic myeloid leukemia; EGFR, Epidermal growth factor receptor; Chemotherapy Facilitation, (1) Primary prophylaxis in patients at high risk for developing febrile neutropenia associated with myelotoxic chemotherapy, (2) Secondary prophylaxis for patients who have experienced neutropenia following prior myelotoxic chemotherapy, (3) To facilitate administration of dose dense chemotherapy regimens; HR, Hormone receptor; HRE2, Human epidermal growth factor receptor 2; NSCLC, Non-small-cell lung carcinoma; WHO-TRS, WHO Technical Report Series, FDA-Approved Label, the U.S. Food and Drug Administration-approved labels.

Zhou Y, *et al. BMJ Glob Health* 2023; 8:e012899. doi: 10.1136/bmjgh-2023-012899

eTable 1 ESMO-MCBS Scores for Cancer Drug Indication Pairs, 2015-2021

Year	Cancer drug indications	Scores on ESMO-MCBS					Status-application year	Final Status
		Lowest	Trial Name	Highest	Trial Name	Whether meet the criterion		
2021	Abemaciclib-HR-positive/ HER2-negative advanced breast cancer	3	MONARCH 3	4	MONARCH 2	Yes	Rejected	Rejected
2021	Atezolizumab-Locally advanced and metastatic NSCLC	5	OAK, IMpower110	5	OAK, IMpower110	Yes	Rejected	Rejected
2021	Dabrafenib + Trametinib-Metastatic melanoma harbouring BRAFV600 mutation	4	COMBI-d	5	COMBI-v	Yes	Rejected	Rejected
2021	Daratumumab-Newly diagnosed and relapsed/refractory multiple myeloma	Not found		Not found		Not found	Rejected	Rejected
2021	Durvalumab-Locally advanced and metastatic NSCLC	4	PACIFIC	4	PACIFIC	Yes	Rejected	Rejected
2021	Encorafenib + Binimetinib-Metastatic melanoma harbouring BRAFV600 mutation	A 5	COLUMBUS	A 5	COLUMBUS	Yes	Rejected	Rejected
2021	Everolimus-Subependymal giant cell astrocytoma	Not found		Not found		Not found	Recommended	Recommended
2021	Ibrutinib-Relapsed/refractory CLL	Not found		Not found		Not found	Recommended	Recommended
2021	Imatinib-Philadelphia chromosome positive ALL	Not found		Not found		Not found	Recommended	Recommended
2021	Nivolumab-Locally advanced and metastatic NSCLC	5	CheckMate 017, CheckMate 057	5	CheckMate 017, CheckMate 057	Yes	Rejected	Rejected
2021	Osimertinib-EGFR-mutation positive advanced NSCLC	4	FLAURA	4	FLAURA	Yes	Rejected	Rejected
2021	Palbociclib-HR-positive/ HER2-negative advanced breast cancer	3	PALOMA-2	4	PALOMA-3	Yes	Rejected	Rejected
2021	Pembrolizumab-Locally advanced and metastatic NSCLC	4	KEYNOTE-189	5	KEYNOTE-24,	Yes	Rejected	Rejected

Zhou Y, *et al. BMJ Glob Health* 2023; 8:e012899. doi: 10.1136/bmjgh-2023-012899

					KEYNOTE-010			
2021	Pertuzumab-Metastatic HER2-positive breast cancer	4	CLEOPATRA	4	CLEOPATRA	Yes	Rejected	Rejected
2021	Ribociclib-HR-positive/ HER2-negative advanced breast cancer	4	MONALEESA-3	5	MONALEESA-7	Yes	Rejected	Rejected
2021	Tislelizumab-Locally advanced or metastatic urothelial cancer	Not found		Not found		Not found	Rejected	Rejected
2021	Tislelizumab-Relapsed/refractory Hodgkin lymphoma	Not found		Not found		Not found	Rejected	Rejected
2021	Vemurafenib + Cobimetinib-Metastatic melanoma harbouring BRAFV600 mutation	4	coBRIM	4	coBRIM	Yes	Rejected	Rejected
2021	Zanubrutinib-Relapsed/refractory CLL	Not found		Not found		Not found	Rejected	Rejected
2021	Zanubrutinib-Relapsed/refractory mantle cell lymphom	Not found		Not found		Not found	Rejected	Rejected
2019	Afatinib-EGFR mutation-positive advanced NSCLC	4	LUX - Lung 3	4	LUX - Lung 3	Yes	Recommended	Recommended
2019	Atezolizumab-Locally advanced and metastatic NSCLC	5	OAK, IMpower110	5	OAK, IMpower110	Yes	Rejected	Rejected
2019	Bortezomib-Multiple myeloma	Not found		Not found		Not found	Recommended	Recommended
2019	Erlotinib-EGFR mutation-positive advanced NSCLC	4	EURTAC	4	EURTAC	Yes	Recommended	Recommended
2019	Gefitinib-EGFR mutation-positive advanced NSCLC	4	IPASS	4	IPASS	Yes	Recommended	Recommended
2019	Lenalidomide-Multiple myeloma	Not found		Not found		Not found	Recommended	Recommended
2019	Nivolumab-Metastatic melanoma	A 4	CheckMate 066	A 4	CheckMate 066	Yes	Recommended	Recommended
2019	Nivolumab-Locally advanced and metastatic NSCLC	5	CheckMate 017, CheckMate 057	5	CheckMate 017, CheckMate 057	Yes	Rejected	Rejected

2019	Pembrolizumab-Metastatic melanoma	3	KEYNOTE-002	3	KEYNOTE-002	Yes	Recommended	Recommended
2019	Pembrolizumab-Locally advanced and metastatic NSCLC	4	KEYNOTE-189	5	KEYNOTE-24, KEYNOTE-010	Yes	Rejected	Rejected
2019	Pertuzumab-Early stage HER2-positive breast cancer	C	NeoSphere	A	APHINITY	Yes	Rejected	Rejected
2019	Pertuzumab-Metastatic HER2-positive breast cancer	4	CLEOPATRA	4	CLEOPATRA	Yes	Rejected	Rejected
2019	Thalidomide-Multiple myeloma	Not found		Not found		Not found	Recommended	Recommended
2019	Trastuzumab Emtansine-Metastatic breast cancer	4	EMILIA	4	EMILIA	Yes	Rejected	Rejected
2017	Afatinib-EGFR mutation-positive advanced NSCLC	Not available		Not available		Not available	Rejected	Recommended
2017	Crizotinib-ALK-positive metastatic NSCLC	4	PROFILE 1014	4	PROFILE 1014	Yes	Rejected	Rejected
2017	Dasatinib-Imatinib-resistant CML	Not found		Not found		Not found	Recommended	Recommended
2017	Erlotinib-EGFR mutation-positive advanced NSCLC	4	EURTAC	4	EURTAC	Yes	Rejected	Recommended
2017	Gefitinib-EGFR mutation-positive advanced NSCLC	Not available		Not available		Not available	Rejected	Recommended
2017	Nilotinib-Imatinib-resistant CML	Not found		Not found		Not found	Recommended	Recommended
2017	Trastuzumab Emtansine-Metastatic breast cancer	4	EMILIA	4	EMILIA	Yes	Rejected	Rejected
2015	All-Trans Retinoic Acid-Acute promyelocytic leukaemia	Not found		Not found		Not found	Recommended	Recommended
2015	Dasatinib-Imatinib-resistant CML	Not found		Not found		Not found	Rejected	Recommended
2015	Erlotinib-EGFR mutation-positive advanced NSCLC	Not available		Not available		Not available	Rejected	Recommended

Zhou Y, *et al. BMJ Glob Health* 2023; 8:e012899. doi: 10.1136/bmjgh-2023-012899

2015	Filgrastim-Facilitator	Not found; Not available		Not found; Not available		Not found; Not available	Recommended	Recommended
2015	Gefitinib-EGFR mutation-positive advanced NSCLC	Not available		Not available		Not available	Rejected	Recommended
2015	Imatinib-CML	Not found		Not found		Not found	Recommended	Recommended
2015	Imatinib-Gastrointestinal stromal tumour	Not found		Not found		Not found	Recommended	Recommended
2015	Nilotinib-Imatinib-resistant CML	Not found		Not found		Not found	Rejected	Recommended
2015	Rituximab-CLL	Not found		Not found		Not found	Recommended	Recommended
2015	Rituximab-Diffuse large B-cell lymphoma	Not found		Not found		Not found	Recommended	Recommended
2015	Rituximab-Follicular lymphoma	Not found; Not available		Not found; Not available		Not found; Not available	Recommended	Recommended
2015	Trastuzumab-Early stage HER2 positive breast cancer	Not available		Not available		Not available	Recommended	Recommended
2015	Trastuzumab-Metastatic HER2 positive breast cancer	Not found		Not found		Not found	Recommended	Recommended

† Not found: The drug cannot be found on the ESMO-MCBS website; Not available: no trials were cited in WHO Technical Report Series; Letter score, e.g., "A", means score in the curative setting while number, e.g., "4", means score in non-curative setting.

ESMO-MCBS, European Society for Medical Oncology-Magnitude of Clinical Benefit Scale.

ALK, anaplastic lymphoma kinase; ALL, acute lymphoblastic leukaemia; CLL, chronic lymphocytic leukaemia; CML, chronic myeloid leukemia; EGFR, epidermal growth factor receptor; Chemotherapy facilitation, (1) primary prophylaxis in patients at high risk for developing febrile neutropenia associated with myelotoxic chemotherapy, (2) secondary prophylaxis for patients who have experienced neutropenia following prior myelotoxic chemotherapy, (3) to facilitate administration of dose dense chemotherapy regimens; HR, hormone receptor; HER2, human epidermal growth factor receptor 2; NSCLC, Non-small-cell lung cancer

eTable 2 Discrepancies in Documented Evidence of OS Benefit in World Health Organization Technical Report Series and Pivotal Trials Obtained from US Food and Drug Administration-Approved Labels

EML Year	Drug_Indication	Recommended for inclusion in WHO EML	Documented OS benefit†		Discrepancy
			WHO-TRS	FDA-approved label	
2015	Trastuzumab_Early stage HER2 positive breast cancer	Yes	N/A	Yes	WHO-TRS: no OS data mentioned [no reference cited]<br>FDA-approved label: documented OS benefit [clinical trials: doxorubicin and cyclophosphamide followed by paclitaxel (AC→paclitaxel) alone vs paclitaxel plus Herceptin]
2019	Afatinib_EGFR mutation-positive advanced NSCLC	Yes	No	Yes	WHO-TRS: documented lack of OS benefit [clinical trials: afatinib vs chemotherapy]<br>FDA-approved label: documented OS benefit [clinical trials: afatinib vs erlotinib]
2015	Imatinib_Gastrointestinal stromal tumour#	Yes	Yes	No	WHO-TRS: documented OS benefit [clinical trials: different dosing comparison]<br>FDA-approved label: documented lack of OS benefit [clinical trials: different dosing comparison]
2015	Rituximab_CLL	Yes	Yes	No	WHO-TRS: documented OS benefit [a systematic review of RCTs]<br>FDA-approved label: no OS data mentioned
2015	Rituximab_Diffuse large B-cell lymphoma#	Yes	Yes	No	WHO-TRS: documented OS benefit [clinical trials: rituximab+doxorubicin, vincristine, and prednisone (CHOP) vs CHOP]<br>FDA-approved label: documented lack of OS benefit [clinical trials: rituximab+doxorubicin, vincristine, and prednisone (CHOP) vs CHOP]
2015	Rituximab_Follicular lymphoma	Yes	Yes	No	WHO-TRS: documented OS benefit [clinical trials: rituximab+cyclophosphamide, vincristine and prednisone (CVP) vs CVP]<br>FDA-approved label: no OS data mentioned
2015	Trastuzumab_Metastatic HER2 positive breast cancer	Yes	Yes	No	WHO-TRS: documented OS benefit [systematic review of RCTs]<br>FDA-approved label: no OS data mentioned
2017	Crizotinib_ALK-positive metastatic NSCLC	No	Yes	No	WHO-TRS: documented OS benefit [retrospective analysis: crizotinib vs chemotherapy]<br>FDA-approved label: documented lack of OS benefit [the evidence cited were same with the evidence cited by WHO-TRS except that FDA-approved label did not cite evidence from the retrospective analysis]
2019	Erlotinib_EGFR mutation-positive advanced NSCLC#	Yes	Yes	No	WHO-TRS: documented OS benefit [retrospective analysis, TKI→chemo vs chemo→TKI, OS benefit]<br>FDA-approved label: documented lack of OS benefit [clinical trials: erlotinib vs standard chemotherapy]
2019	Gefitinib_EGFR mutation-positive advanced NSCLC#	Yes	Yes	No	WHO-TRS: documented OS benefit [clinical trials: gefitinib monotherapy vs combination therapy with gefitinib, carboplatin, and pemetrexed]<br>FDA-approved label: documented lack of OS benefit [clinical trials: gefitinib vs carboplatin/paclitaxel, OS data reported]
2019	Thalidomide_Multiple myeloma	Yes	Yes	No	WHO-TRS: documented OS benefit [a rapid Cochrane network meta-analysis of RCTs]<br>FDA-approved label: documented lack of OS benefit [clinical trials: Thalidomide/Dexamethasone vs placebo/Dexamethasone]
2015	All-Trans Retinoic Acid_Acute promyelocytic leukaemia	Yes	Yes	N/A	WHO-TRS: documented OS benefit [clinical trials: two courses of chemotherapy vs three courses of chemotherapy]<br>FDA-approved label: no data [label not found]
2021	Imatinib_Philadelphia chromosome positive ALL	Yes	Yes	No	WHO-TRS: documented OS benefit [systematic review of eight comparative cohort studies]<br>FDA-approved label: no OS data mentioned

†: No documented OS benefit includes three scenarios: (a) lack of OS benefit, i.e., WHO-TRS/FDA-approved label mentioned OS but there was no statistically significant OS benefit; (b) no OS data mentioned, i.e., WHO-TRS/FDA-approved label did not report any information on OS; (c) the label was unavailable or the drug was not approved by FDA, e.g., a label for all-Trans Retinoic Acid could not be found, and Tislelizumab (EML decision year 2021) is not approved by FDA.
#: Targeted cancer drug indications with conflicting evidence: WHO-TRS documented OS benefit while at the time of WHO EML decision, the trial results documented in FDA-approved labels were not statistically significant.
OS, Overall survival; WHO-TRS, WHO Technical Report Series.

Zhou Y*, et al. BMJ Glob Health* 2023; 8:e012899. doi: 10.1136/bmjgh-2023-012899
